# Pantoprazole as a potential repurposed drug targeting the adenosinergic system in esophageal cancer

**DOI:** 10.1007/s00044-026-03618-2

**Published:** 2026-09-25

**Authors:** Adriana Simioni, Rafael D Weimer, Julia B de Souza, Isadora V Citrini, Julia A da Rosa, Kaline Cardoso, Maria E Delawi, Luis FSM Timmers, Fernanda B Morrone

**Affiliations:** 1 Laboratório de Farmacologia Aplicada, Escola de Ciências da Saúde e da Vida, PUCRS, Porto Alegre, RS Brasil; 2 Programa de Pós-graduação em Biologia Celular e Molecular, Escola de Ciências da Saúde e da Vida, PUCRS, Porto Alegre, RS Brasil; 3 Programa de Pós-graduação em Medicina e Ciências da Saúde, Escola de Medicina, PUCRS, Porto Alegre, RS Brasil; 4 Programa de Pós-graduação em Biotecnologia, Univates, Lajeado, RS Brasil; 5 Programa de Pós-graduação em Ciências Médicas, Univates, Lajeado, RS Brasil

**Keywords:** Pantoprazole, Adenosinergic system, P1 receptors, Esophageal cancer, Drug repurposing

## Abstract

Esophageal cancer remains a major global health burden and is associated with poor prognosis. Adenosine signaling, particularly through CD73 and P1 receptors, contributes to tumor progression and immune modulation. Pantoprazole, a proton pump inhibitor widely used in clinical practice, has recently been suggested as a potential modulator of adenosinergic pathways. This study investigated the ability of pantoprazole to modulate adenosine signaling. In silico docking analyses were performed to assess the binding of pantoprazole to adenosine P1 receptors and CD73, followed by in vitro assays using esophageal adenocarcinoma (OE33) and squamous cell carcinoma (KYSE450) cell lines to evaluate cell growth, adenosinergic signaling, and tumor-related cellular functions following pantoprazole treatment. Docking analyses identified CD73 and P1 receptors, particularly A_2A_, as favorable molecular targets for pantoprazole. Pantoprazole significantly reduced proliferation and metabolic activity in both cell lines in a concentration-dependent manner, with pronounced inhibition of clonogenic survival in adenocarcinoma cells. Pantoprazole altered P1 receptor expression, particularly A_2A_, and pharmacological antagonism supported the involvement of these receptors in its antiproliferative effects. Additionally, pantoprazole modulated CD73 expression and reduced cell adhesion and MMP‑2 expression. These findings indicate that pantoprazole exerts antitumor effects by modulating the CD73–adenosine–P1 receptor axis, supporting its potential repurposing as an adjunctive strategy for the treatment of esophageal adenocarcinoma.

**Supplementary Information:**

The online version contains supplementary material available at https://doi.org/10.1007/s00044-026-03618-2.

**Figure:**
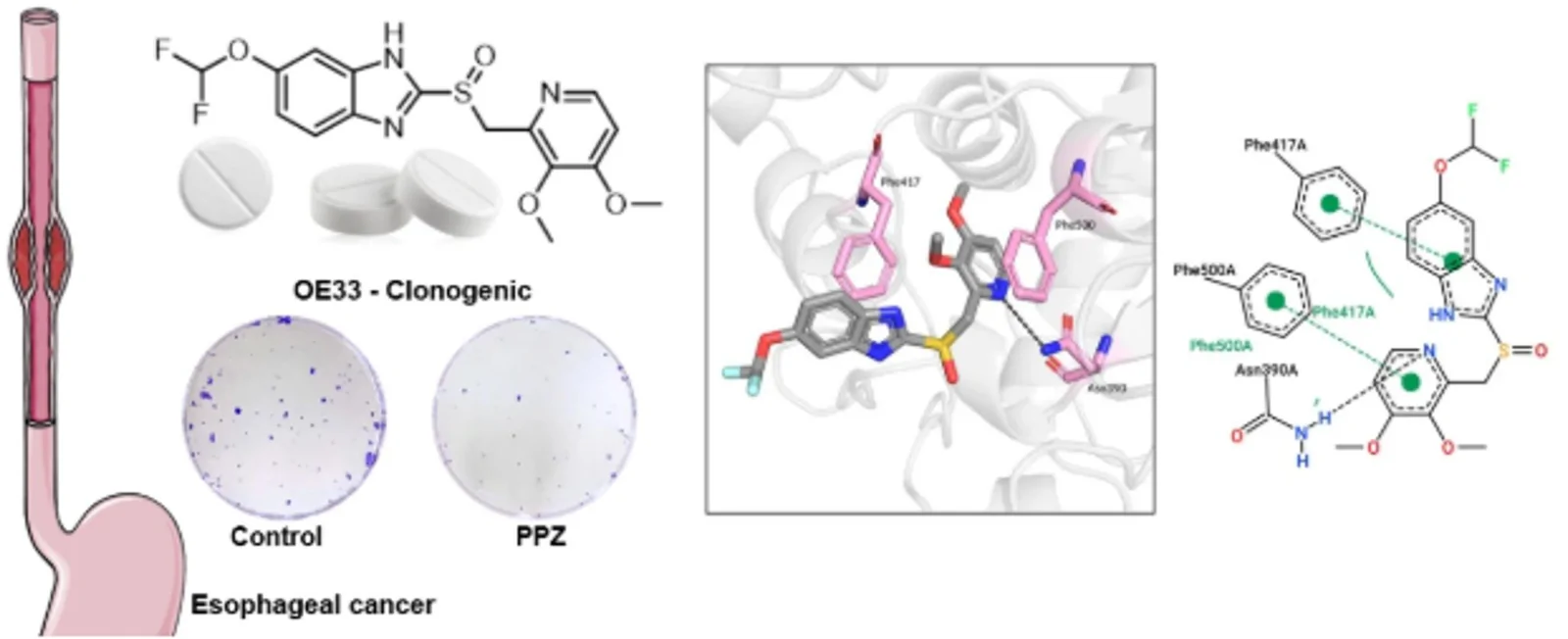

## Introduction

Esophageal carcinoma is a disease with high morbidity and mortality, diagnosed at an advanced stage due to the absence of initial signs [1]. Its pathogenesis is multifactorial, involving oxidative stress, reflux-induced inflammation, and chronic esophagitis. These conditions drive a high rate of cellular turnover alongside genetic and epigenetic alterations, such as the aberrant methylation of promoter regions, which activate oncogenes and silence tumor suppressor genes. Risk factors include cultural habits, such as the consumption of mate tea in high temperatures and a higher intake of red meat in southern Brazil, Argentina, and Uruguay [2], as well as smoking, alcoholism, and an association with *Helicobacter pylori* infection [3]. There are two main tumor subtypes: squamous cell carcinoma (SCC), which is primarily related to lifestyle habits and arises in the upper and middle esophagus, and adenocarcinoma (AC), which is more closely related to gastroesophageal reflux disease (GERD) and Barrett’s esophagus (BE), arising predominantly at the gastroesophageal junction [4]. The main treatment is surgical resection, and chemotherapy or chemoradiotherapy are used to contain tumor growth [5].

Nucleotides and nucleosides regulate various cellular functions [6], and studies have indicated their involvement in different neoplasms, including esophageal carcinoma [7–10]. Extracellular purines (ATP, ADP, and adenosine) and pyrimidines (UTP and UDP) can act as endogenous signaling molecules, exerting effects on the inflammatory and immune response, neurotransmission, muscle contraction, and pain, among others [11].

Adenosine (ADO) is produced both intracellularly and extracellularly. Generally, adenosine concentration is low in the extracellular environment and increases under conditions of metabolic stress, such as inflammation and cancer [12]. ATP and ADP are converted to AMP by the enzyme ecto-nucleoside triphosphate diphosphohydrolase (E-NTPDase, CD39), and then ecto-5’-nucleotidase (ecto-5’-NT, CD73) hydrolyzes AMP into adenosine [13]. Adenosine can be metabolized to inosine by the enzyme adenosine deaminase (ADA) and subsequently to hypoxanthine, and can also be converted to uric acid by xanthine oxidase. Adenosine can also be reconverted to 5’-AMP by the enzyme adenosine kinase and then to ADP and ATP [14].

Adenosine (ADO) is involved in multiple metabolic pathways and exerts its biological effects through two families of purinergic receptors, P1 and P2 receptors [11]. A previous study by Santos et al. (2017) [10] demonstrated that high concentrations of ATP reduced cell number, viability, colony formation, and migration in esophageal squamous cell carcinoma cell lines. The same study showed that ATP activates P2X7 receptors, suggesting that the observed cytotoxic effects may be mediated through P2X7x-dependent signaling pathways. In addition, P2Y receptors are expressed in esophageal cancer cells, and their activation by ATP increases intracellular calcium levels through phospholipase C signaling. This pathway plays an important role in the antiproliferative effects of ATP by promoting apoptosis and inducing cell cycle arrest [15]. Once in the extracellular environment, mainly after AMP hydrolysis, adenosine triggers its numerous functions by activating the class of receptors P1 [16].

P1 receptors are metabotropic G protein-coupled receptors and are divided into subtypes A_1_, A_2A_, A_2B_, and A_3_ [17]. They differ in their affinity for adenosine, the type of G protein recruited, and the signaling pathways activated: A_1_, A_2A_, and A_3_ have high affinity, while A_2B_ has low affinity [18] A_1_ and A_3_, coupled to Gi/o, inhibit adenylate cyclase, reducing cyclic AMP and favoring immune responses, while A_2A_ and A_2B_, coupled to Gs, increase cyclic AMP, promoting immunosuppression [19]. These receptors can activate MAPK pathways, related to processes such as inflammation, apoptosis, and cell proliferation [16].

The development of new drugs is a long and complex process, with high costs and high failure rates. In this context, drug repurposing emerges as a promising strategy, allowing the use of already approved drugs for new indications, reducing development time, costs, and risks [20]. Among the candidates, proton pump inhibitors (PPIs) stand out, which act by covalent bonding between SH and cysteine residues in the alpha subunit of H⁺/K⁺-ATPase, irreversibly inhibiting gastric acid secretion [21].

Proton pump inhibitors decrease daily acid production and are used to reduce symptoms and promote healing of gastric and duodenal ulcers and to treat gastroesophageal reflux disease (GERD). In rodents, an increase in extracellular adenosine decreases gastric acid production through A_1_ and A_2A_ receptors [22]. Another study demonstrated that omeprazole decreases the expression of these receptors, inducing a reduction in gastric secretion as a secondary effect through the body’s own regulatory feedback mechanism in response to changes in the acidic environment [23]. Furthermore, lansoprazole induced apoptosis in breast cancer cells by inhibiting proton extrusion [24]. The link between PPI use and gastric neuroendocrine tumors (NETs), gastric adenocarcinomas, and gastric cancer progression from Barrett’s esophagus has been investigated due to hypergastrinemia. In fact, PPIs cause a hypergastrinemia state, increasing enterochromaffin-like cell dysplasia and the risk of developing gastric NETs, increasing the severity of gastritis related to *Helicobacter pylori* infection, as well as the progression of carcinogenesis in predisposed patients due to Barrett’s esophagus [25].

Recent studies indicate that PPIs exhibit antitumor activity in various types of cancer, which have been associated with the induction of apoptosis, modulation of autophagy, and alteration of the acidic tumor microenvironment, making it less favorable for cell survival [26]. A study evaluated the use of esomeprazole in BE-derived adenocarcinoma cells, where esomeprazole significantly inhibited cell proliferation and invasiveness and induced apoptosis in AC tumor cells, without affecting apoptosis in non-dysplastic Barrett’s esophagus cells, showing that the clinical use of the drug in both conditions may be favorable [27]. Besides, pantoprazole was shown to exert an antitumor effect on gastric cancer stem cells, promoting a selective anticancer action by inhibiting tumor progression via the suppression of the EMT and β-catenin pathways. The inhibition of proliferation and a decrease in self-renewal capacity were observed alongside an increase in chemosensitivity to 5-FU, demonstrating a promising therapeutic strategy [28]. Additionally, the use of PPIs in SCC also proved favorable, where after the use of omeprazole, a downregulation was observed in the expression levels of the proteins AHR and PCNA—associated with proliferation—and MMP9—associated with invasion [29]. In addition, PPI therapy can influence the expression of P1 purinergic receptors, especially A_1_ and A_2A_, suggesting a relevant role in the regulation of cellular pathways involved in tumor progression [23]. Given the widespread use of the PPI pantoprazole (PPZ) in the treatment of GERD and the lack of studies on P1 receptors in esophageal cancer, it is of great interest to investigate the action of this drug on the adenosinergic system in the growth of esophageal cancer cells.

## Results

The Boltz-2 model predicts protein–ligand proximity and provides the log10(IC_50_) value, from which the IC₅₀ (µM) for each compound can be calculated based on its predicted binding affinity to the target site [30]. The estimated IC_50_ values (Table 1) for pantoprazole in complex with CD73 (6.109 µM), A_2A_ receptor (3.467 µM), and A_3_ receptor (3.427 µM) stand out from the other tested proteins. According to these results, targets CD73, A_2A,_ and A_3_ were the most favorable to maintain interaction with pantoprazole.

**Table 1:** Boltz-2–predicted IC₅₀ values for pantoprazole-protein complexes

Target protein	log10(IC_50_)	Estimated IC_50_ (µM)
CD73	**0.786**	**6.109**
A_1_ receptor	1.081	12.050
A_2A_ receptor	**0.540**	**3.467**
A_2B_ receptor	1.040	10.964
A_3_ receptor	**0.535**	**3.427**

**Fig. 1:**
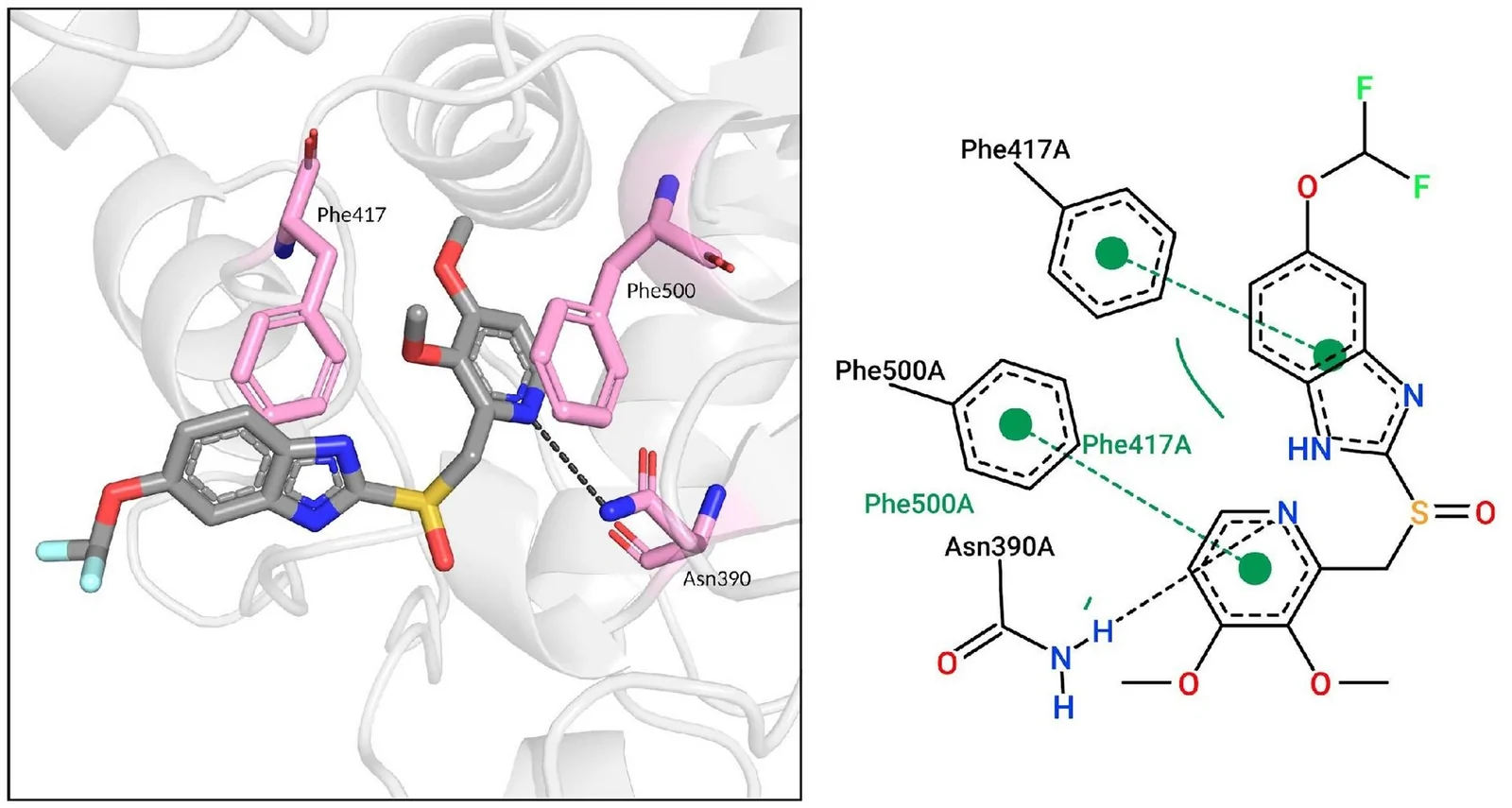
Interactions between PPZ and the CD73 protein. The 3D representation illustrates the interactions with CD73 residues, while the 2D diagram depicts each type of interaction. Interacting residues are shown as pink sticks. Intermolecular hydrogen bonds are indicated by black dashed lines

Analysis of the CD73–PPZ complex (Fig. 1) indicated that PPZ establishes π–π stacking interactions with residues Phe417 and Phe500, involving the aromatic rings of the ligand and the phenylalanine side chains. A hydrogen bond was also identified between PPZ and Asn390, involving the amide group of the residue.

**Fig. 2:**
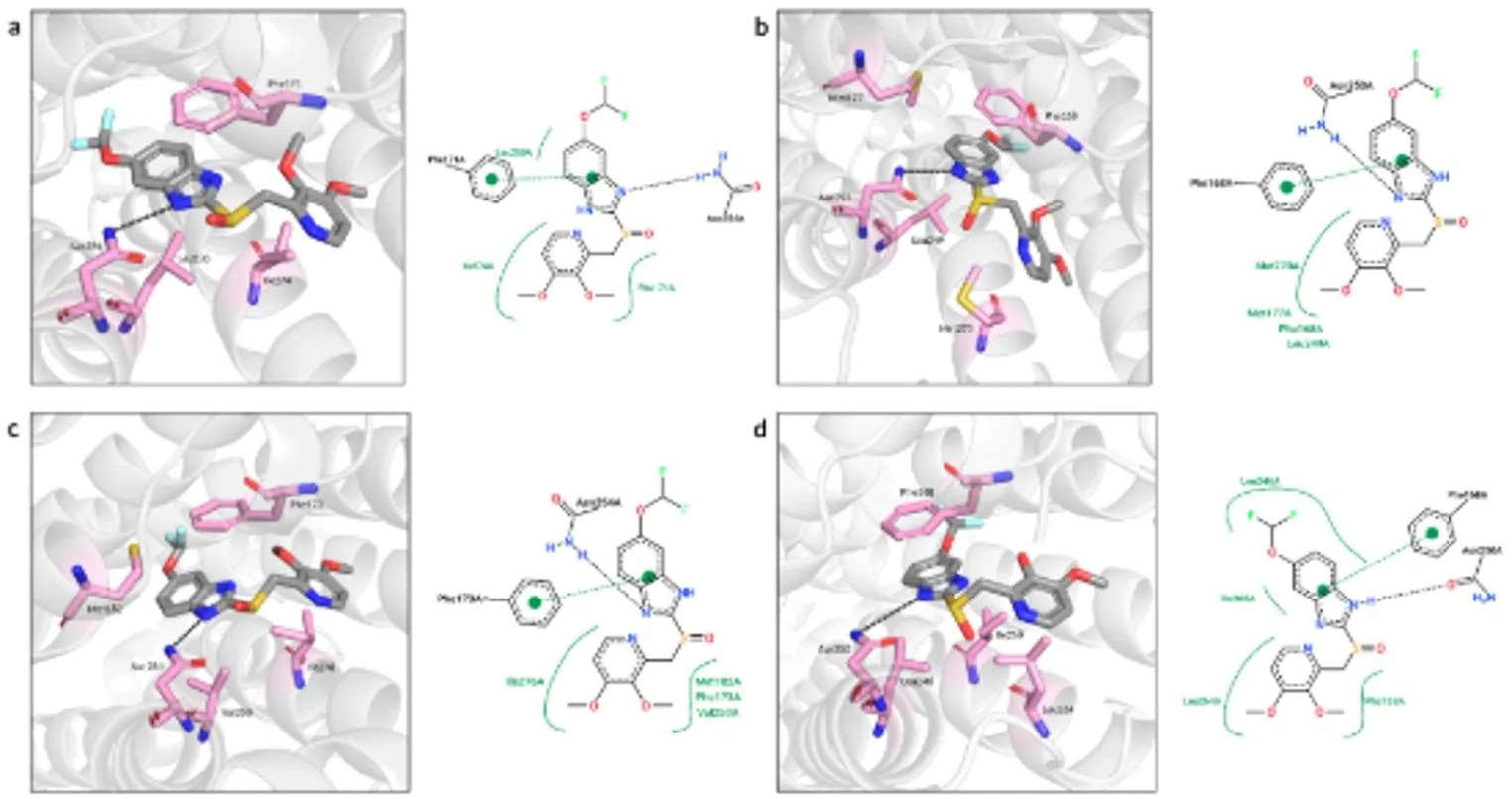
Interactions between PPZ and adenosine receptors.** (a)** A_1_, **(b)** A_2A_, **(c)** A_2B_, **(d)** A_3_. The 3D representations illustrate interactions with receptor residues, while the 2D diagrams depict each interaction type. Interacting residues are shown as pink sticks. Black dashed lines indicate intermolecular hydrogen bonds

For the P1 adenosine receptor complexes, all docking poses revealed a conserved binding pattern, characterized by π–π stacking interactions between phenylalanine residues and the aromatic moiety of PPZ, as well as a hydrogen bond between an asparagine residue and a nitrogen atom of the ligand, similar to what was observed for CD73. Structural superposition further indicated that both the phenylalanine and asparagine residues occupy equivalent positions across all four P1 receptors (A_1_, A_2A_, A_2B_, and A_3_). In addition, hydrophobic contacts between PPZ and the binding pocket were observed in all P1 receptor subtypes. Although the specific residue identities varied among A_1_, A_2A_, A_2B_, and A_3_, these residues occupied equivalent spatial positions within the pocket (Fig. 2). To confirm these results, we also analysed the structural superposition of the predicted models with experimental structures of adenosine targets and docking of PPZ into the orthosteric binding site (Sup. Fig. S1).

**Fig. 3:**
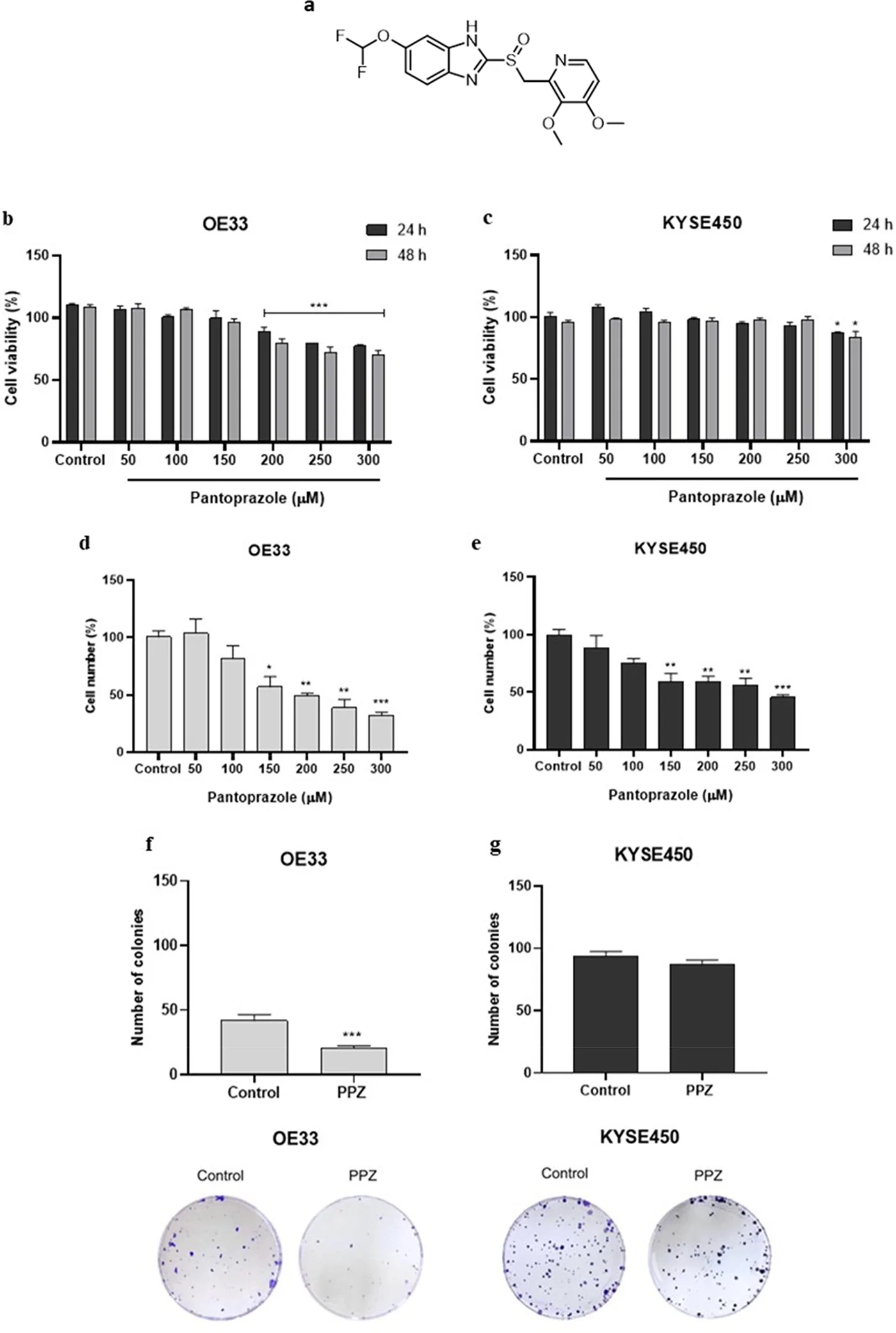
Effect of PPZ on esophageal cell proliferation. **(a)** Chemical structure of PPZ. Effects of PPZ (50–300 µM) on the viability of esophageal cancer cell lines **(b)** OE33 and **(c)** KYSE450, assessed by MTS assay after 24 h and 48 h of treatment. **(d**,** e)** The cell number was determined by a Trypan Blue exclusion assay following PPZ treatment (50–300 µM) for 24 h. **(f**,** g)** Representative images and quantitative analysis of clonogenic assays performed in OE33 and KYSE450 cells after treatment with 150 µM of PPZ. Panels **b** and **c** were analyzed by two-way ANOVA followed by Tukey’s post hoc test, whereas panels **d** and **e** were analyzed by one-way ANOVA followed by Tukey’s post hoc test, and Student’s *t*-test was used to analyze panels **f** and **g.** Data are presented as mean ± SEM. *p < 0.05; **p < 0.01; ***p < 0.001 versus control

To validate the in silico findings, we performed in vitro experiments using two esophageal cancer cell lines representative of adenocarcinoma (OE33) and squamous cell carcinoma (KYSE450). PPZ treatment (Fig. 3a) significantly reduced cell proliferation in both cell lines in a concentration‑ and time‑dependent manner. As shown in Fig. 3b, MTS assays demonstrated that PPZ at 200–300 µM reduced the metabolic activity of OE33 cells in a dose- and time-dependent manner. After 24 h of treatment, metabolic activity decreased to 89.08 ± 3.13%, 79.21 ± 0.35%, and 77.14 ± 1.23% at 200, 250, and 300 µM, respectively. After 48 h, a further reduction was observed, with metabolic activity reaching 79.85 ± 3.27%, 72.40 ± 4.02%, and 70.31 ± 3.34% at the same concentrations. In KYSE450 cells, a significant reduction in metabolic activity was observed only at the highest concentration of PPZ (300 µM) at both time points. After 24 h of treatment, metabolic activity decreased to 87.14 ± 0.99%, while after 48 h it reached 83.57 ± 4.92%, compared to control cells (Fig. 3c).

Cell number assessment by Trypan Blue exclusion revealed a significant reduction in OE33 cells following treatment with PPZ (150–300 µM) for 24 h. Cell numbers decreased to 56.96 ± 9.01%, 49.71 ± 1.87%, 38.92 ± 7.11%, and 32.44 ± 2.51% at 150, 200, 250, and 300 µM, respectively, compared to control cells (Fig. 3d). In KYSE450 cells, treatment with PPZ (150–300 µM) reduced cell numbers after 24 h, yielding values of 58.88 ± 7.41%, 59.22 ± 4.63%, 56.49 ± 5.56%, and 45.44 ± 2.28% at 150, 200, 250, and 300 µM, respectively (Fig. 3e). Based on these results, a concentration of 150 µM was selected for all subsequent experiments.

To evaluate the long-term effects of PPZ on the ability of a single cell to survive and form colonies, a clonogenic assay was performed in both esophageal cancer cells. Cells were exposed to PPZ for 24 h and then re-plated at low density in drug-free medium. PPZ treatment markedly impaired the clonogenic capacity of adenocarcinoma OE33 cells. After 10 days, colony formation was reduced to 20.75 ± 1.63 colonies, representing an approximately 50% decrease compared with control cells (Fig. 3f).

**Fig. 4:**
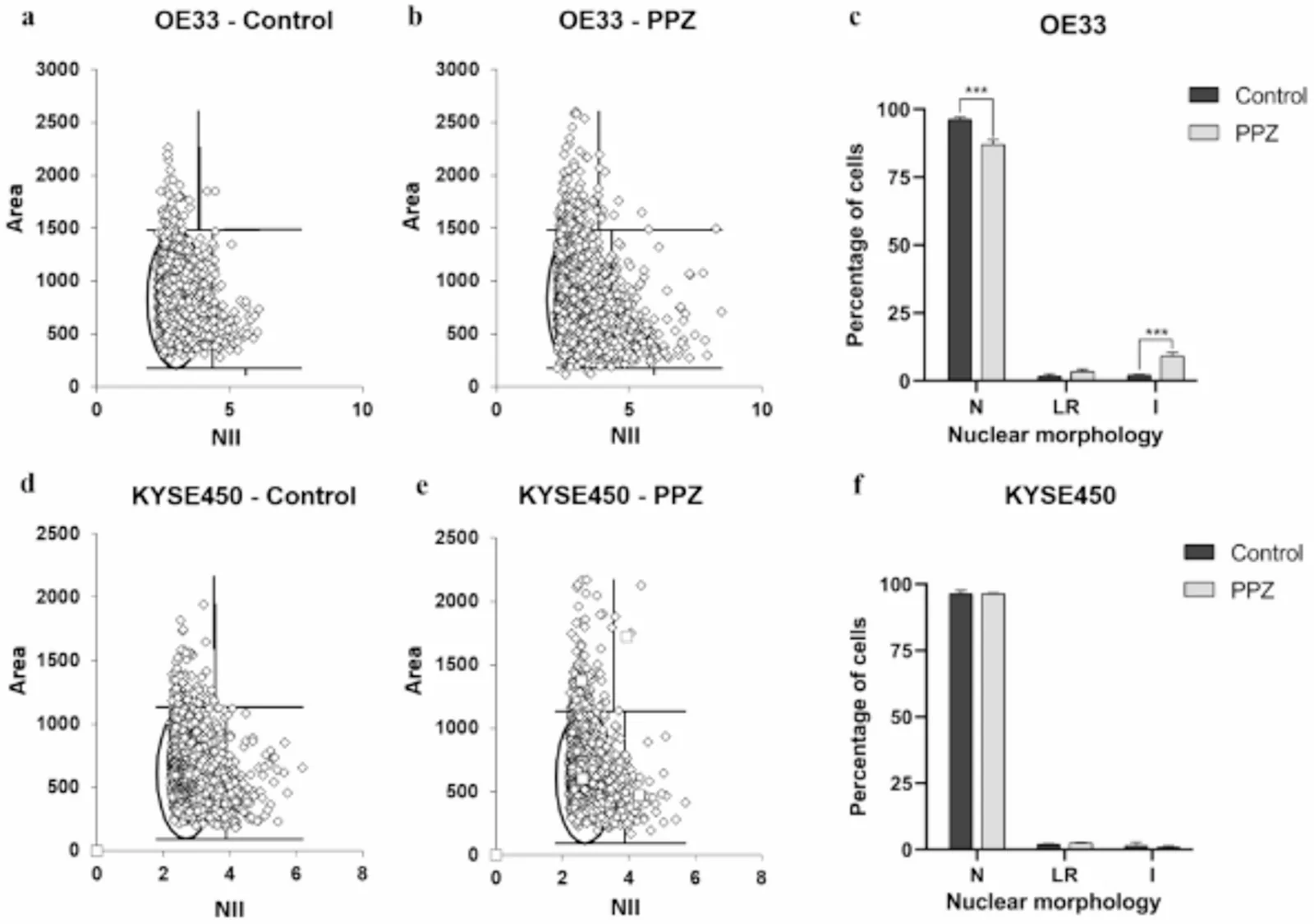
Nuclear morphology analysis (NMA) after PPZ treatment. OE33 and KYSE450 cells were treated with PPZ (150 µM) for 24 h. Dispersion plots of nuclear morphology distribution based on the Nuclear Irregularity Index (NII) of **(a**,** b)** OE33 and **(d**,** e)** KYSE450 control groups and after 24 h of PPZ treatment. Nuclei were classified into distinct populations according to nuclear area and regularity: N, normal nucleus; I, irregular nucleus; LR, large and regular nucleus; LI, large and irregular nucleus; SR, small and regular nucleus; S, small nucleus; and SI, small and irregular nucleus. **(c**,** f)** Graphical representation of nuclear populations of OE33 and KYSE450 cells. Panels **c** and **f** were analyzed by Student’s *t*-test. Data are presented as mean ± SEM. ***p < 0.001 versus control

In addition, nuclear morphology analysis (NMA) of the surviving tumor cell populations after 24 h of exposure to PPZ revealed alterations in nuclear morphology. As shown in Fig. 4, PPZ‑treated OE33 adenocarcinoma cells exhibited marked nuclear alterations compared with vehicle‑treated controls, including a significant increase in the irregular (I) population (Fig. 4a-c). In contrast, KYSE450 cells did not exhibit significant alterations in nuclear morphology following treatment (Fig. 4d-e). Notably, a subset of PPZ-treated OE33 cells displayed markedly condensed, elongated nuclei, as indicated by the red arrows in Supplementary Figure S2, a characteristic not observed in controls or PPZ-treated KYSE450 cells.

To complement the NMA, we quantified cell death by annexin V/PI staining (Sup. Fig. S3). PPZ treatment produced a small but statistically significant increase in the early apoptotic population in adenocarcinoma OE33 cells, whereas the late apoptotic/necrotic population fraction remained unchanged. In KYSE450 cells, both early apoptotic and the late apoptotic/necrotic populations were significantly increased following treatment, although the magnitude of these changes was likewise limited. Taken together, these data indicate that PPZ induces only a modest apoptotic response in both cell lines at 24 h of treatment, which is insufficient to account for the observed reduction in cell numbers and suggests that apoptosis is not the main mechanism underlying the cellular response to PPZ.

**Fig. 5:**
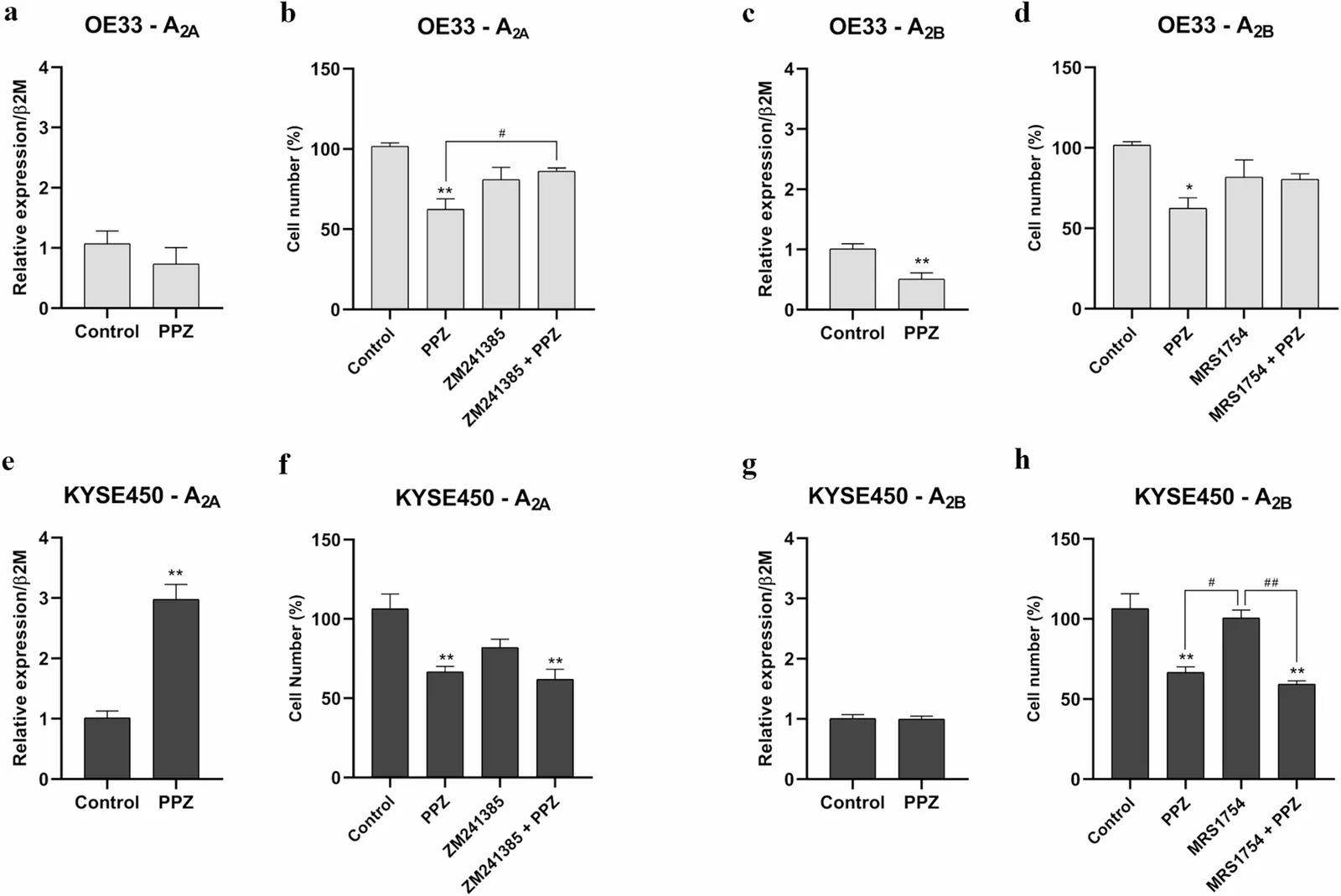
Adenosine receptor expression and effect of selective A_2A_/A_2B_ antagonists on PPZ-induced proliferation inhibition in esophageal cancer cells. Relative mRNA expression levels of A_2A_ and A_2B_ receptors after treatment with PPZ (150 µM) in **(a**,** c)** OE33 and (**e**,** f)** KYSE450. **(b)** OE33 and **(f)** KYSE450 cell numbers were assessed after treatment with PPZ (150 µM), A_2A_ antagonist (1 µM, ZM241385) or PPZ in combination with the A_2A_ antagonist. **(d)** OE33 and **(h)** KYSE450 cell numbers were assessed after treatment with PPZ (150 µM), A_2B_ antagonist (1 µM, MRS1754) or PPZ in combination with the A_2B_ antagonists. Each column represents the mean ± SEM. Panels **a**, **c**, **e** and **g** were analyzed by Student’s *t*-test, and panels **b**, **d**, **f** and **h** were analyzed by one-way ANOVA followed by Tukey’s post-hoc test. (*) represents the statistical differences relative to the control group, where *p < 0.05, **p < 0.01, and ***p < 0.001. (#) represents statistical differences relative to the MRS1754 group, where #*p* < 0.05 and ##*p* < 0.01

Afterward, to investigate the mechanisms underlying the response to PPZ treatment, we analyzed the expression of P1 receptors in KYSE-450 squamous cell carcinoma and in O33 adenocarcinoma cells. Notably, according to the Human Gene Database [31], both esophageal cancer cell lines used in this study lack A_1_ receptor expression and exhibit very low A_3_ receptor expression levels. Consistent with these data, RT‑qPCR analysis confirmed the expression of A_2A_ and A_2B_ receptors in both cell lines studied. Interestingly, PPZ treatment reduced A_2A_ and A_2B_ receptor expression in adenocarcinoma cells (Fig. 5a and c). In contrast, in squamous carcinoma cells, PPZ increased A_2A_ mRNA expression, while no significant change was observed in A_2B_ receptor expression (Fig. 5e and g). We further investigated the effects of selective P1 receptor antagonists targeting A_2A_ receptor (ZM 241385) and A_2B_ receptor (MRS 1754). Treatment with selective P1 receptor antagonists alone did not significantly alter cell number in the conditions tested (Fig. 5). Functional assays demonstrated that PPZ (150 µM) reduced cell numbers in both cell lines; however, co‑treatment with A_2A_ and A_2B_ receptor antagonists (ZM 241385 and MRS 1754, respectively) reversed this effect only in adenocarcinoma cells (Fig. 5b and d). These findings support the involvement of A_2A_ and A_2B_ receptor signaling in the antiproliferative effects of PPZ in this tumor subtype.

**Fig. 6:**
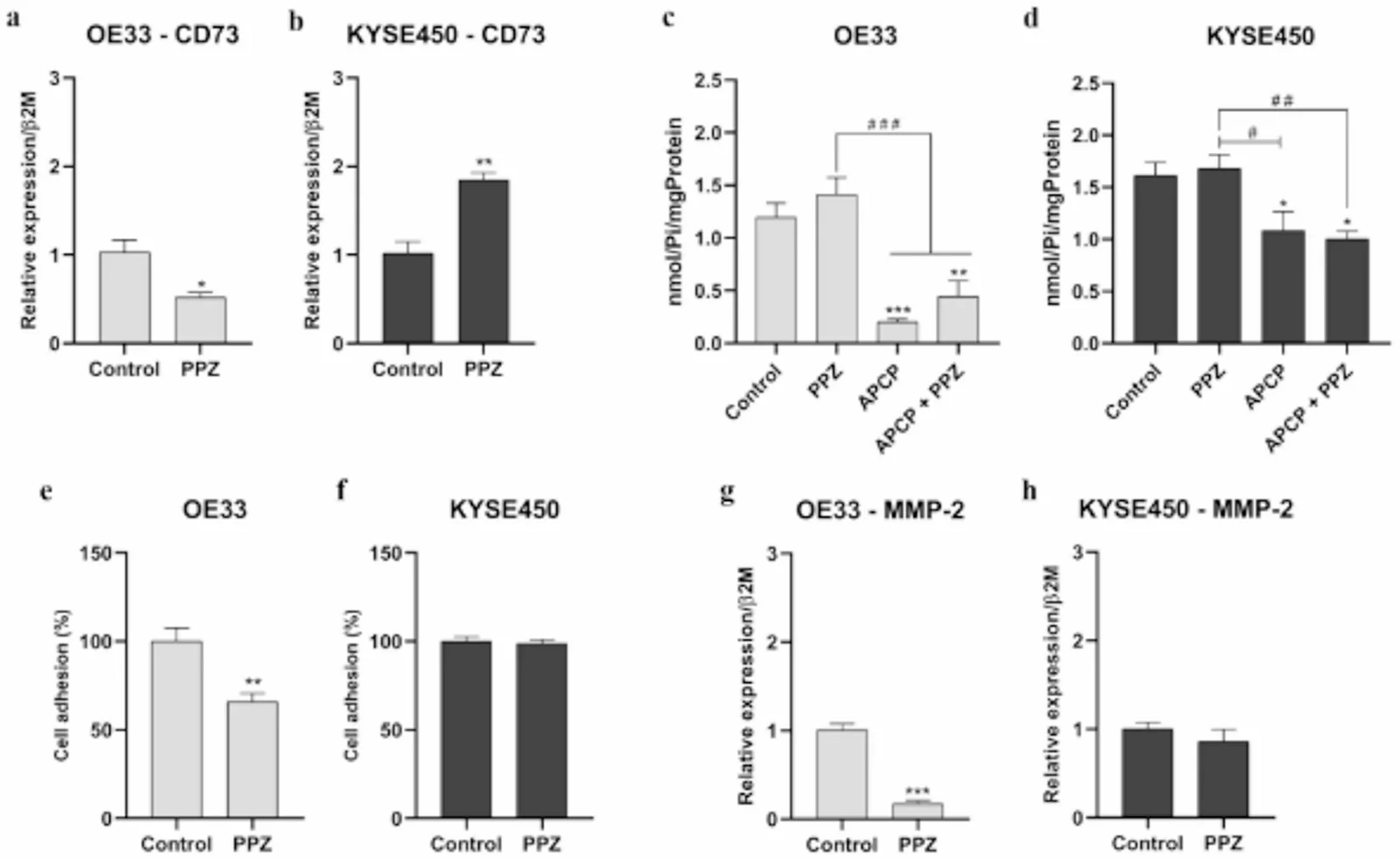
Effect of PPZ on CD73 and esophageal cell adhesion.** (a**,** b)** mRNA expression levels of CD73 in OE33 and KYSE450 cells after treatment with PPZ (150 µM). **(c**,** d)** AMPase enzymatic activity in esophageal cancer cells. OE33 and KYSE450 cells were treated with PPZ (150 µM) for 24 h. AMP hydrolysis was quantified using the Malachite Green assay. Where indicated, cells were treated with APCP, a CD73 inhibitor. **(e**,** f)** For the adherence assay, the cells were treated with PPZ (150 µM) and, after 24 h, were replated to adhere for 3 h. **(g**,** h)** mRNA expression levels of MMP-2 in OE33 and KYSE450 cells after treatment with PPZ (150 µM). Panels **a**,** b**,** e-h** were analyzed by Student’s *t*-test, and panels **c** and **d** were analyzed by one-way ANOVA followed by Tukey’s post hoc test. *p < 0.05; **p < 0.01

Following docking analysis of the CD73–PPZ complex, we evaluated CD73 expression and enzymatic activity after PPZ treatment. As shown in Fig. 6a and b, PPZ reduced CD73 expression in the adenocarcinoma cell line OE33, whereas CD73 expression increased in the squamous cell carcinoma cell line. PPZ at 150 µM did not significantly alter AMP hydrolysis in either cell line (Fig. 6c and d). Treatment with APCP, a CD73 inhibitor, significantly reduced AMP hydrolysis. Moreover, cotreatment with PPZ and APCP further decreased AMP hydrolysis in both cell lines. In OE33 cells, the PPZ + APCP group showed significantly lower AMP hydrolysis compared with the control (0.441 ± 0.147 vs. 1.196 ± 0.133 nmol Pi/mg protein) and PPZ alone (1.414 ± 0.159 nmol Pi/mg protein). Similarly, in KYSE450 cells, the PPZ + APCP group exhibited reduced AMP hydrolysis (1.003 ± 0.075 nmol Pi/mg protein) compared with the control (1.613 ± 0.125 nmol Pi/mg protein) and PPZ alone (1.683 ± 0.130 nmol Pi/mg protein).

Since the adenosine-CD73 pathway is involved in cell adhesion, we evaluated the effect of PPZ on the adhesion of the esophageal cells and on the expression of the metalloproteinase 2 (MMP-2). Interestingly, treatment with PPZ altered the adhesion of adenocarcinoma cells OE33 and diminished the expression of MMP-2 in these cells (Fig. 6e and g). The same effect was not observed in the squamous cell line KYSE450 (Fig. 6f and h).

## Discussion

In recent decades, the use of PPIs, particularly PPZ, has increased substantially. Although these agents are widely prescribed for acid‑related disorders, their expanding use has raised concerns regarding adverse effects, drug interactions, and inappropriate long‑term exposure [32]. In addition, suppression of gastric acid secretion compromises an important physiological barrier, potentially increasing susceptibility to infections and other complications [22]. Beyond their gastric effects, accumulating evidence suggests that PPIs may influence tumor‑related pathways. In this context, the present study investigated the interaction of PPZ with the adenosinergic system, specifically, P1 receptors and CD73, and its functional consequences in esophageal cancer models.

Docking experiments showed that PPZ established π–π stacking interactions with Phe417 and Phe500 of CD73, which have previously been reported as key components of AMP hydrolysis. These residues form the adenine clamp, a structural feature that stabilizes the adenine nucleobase through hydrophobic π-stacking interactions. The interaction of PPZ with these aromatic residues suggests potential interference with substrate binding [33]. In addition, Asn390, known to be involved in ribose positioning, forms a hydrogen bond with the ligand, further supporting its relevance for inhibitor binding, as also reported by [34].

Structural insights from the A_1_ [35], A_2A_ [36], A_2B_ [37], and A_3_ [38] adenosine receptors support our docking analysis results. These studies have shown that ligand recognition in P1 receptors relies on conserved aromatic residues and a key asparagine, which mediate π–π stacking and hydrogen bonding interactions, respectively. This conserved binding pattern suggests that PPZ may exploit common recognition motifs of the adenosinergic system, potentially interfering with adenosine signaling at multiple levels. Despite minor differences in residue identity, the hydrophobic environment surrounding PPZ was preserved across all P1 receptors, supporting a conserved binding mode.

Purinergic signaling, altered in various tumors, is associated with increased extracellular adenosine, which favors cancer development through its role in the immune response [39]. Adenosine modulates physiological and pathological processes, including angiogenesis, proliferation, and apoptosis [11]. It promotes wound healing and angiogenesis via A_2A_ and A_2B_ receptors, which are fundamental for tumor growth and metastasis, but it can also reduce tumor growth through apoptosis mediated by A_1_, A_2A_, and A_3_ receptors [40].

Supporting evidence from [23] showed that adenosine inhibits immunoreactive gastrin release and stimulates somatostatin immunoreactivity through activation of A_1_ and A_2A_ receptors. Similarly, Redzic et al. [22] reported that omeprazole-induced achlorhydria modified nucleoside transporter expression in rat gastric mucosa, potentially contributing to purinergic feedback regulation of acid secretion. Omeprazole-induced achlorhydria suppressed A_1_ and A_2A_ receptor gene expression in the antrum and body mucosa, but not in body muscle, suggesting that PPI treatment may alter P1 receptor expression and function. Other studies have shown that rabeprazole decreases gastric cancer cells’ viability, indicating its potential as an antineoplastic agent [41].

Functionally, PPZ reduced proliferation and viability of both OE33 adenocarcinoma and KYSE450 squamous carcinoma cells in a concentration‑ and time‑dependent manner. However, long‑term clonogenic survival and nuclear morphology analyses revealed a marked histology‑dependent response. PPZ significantly impaired clonogenic capacity and altered nuclear morphology selectively in OE33 cells, whereas KYSE450 cells exhibited minimal morphological changes. Overall, these findings suggest that adenocarcinoma cells are more susceptible to PPZ treatment. The limited apoptotic response and the pronounced reduction in the long-term colony formation, together with the increase in the irregular nuclei observed in NMA indicate that PPZ may interfere with cell-cycle progression or with other processes affecting proliferative capacity, although this requires further investigations. V-ATPase expression in cervical adenocarcinoma correlates with poor prognosis, and its inhibition via siRNA or PPIs (esomeprazole) enhances paclitaxel chemosensitivity [42]. Accordingly, Zhang et al. [24] demonstrated that lansoprazole induces dose-dependent apoptosis in breast cancer cells, inhibits proton extrusion, and triggers lysosomal alkalinization and ROS accumulation, suggesting its potential as an anticancer agent.

Adenosine regulates innate and adaptive immune responses, and its dysregulation is associated with diseases such as cancer, epilepsy, and psychiatric disorders [43]. The differential expression of adenosine receptors in normal and tumor cells suggests their potential as biomarkers and therapeutic targets [11]. Several effects of P1 receptors have been described in cancers such as colon, breast, gliomas, and melanomas, but there are no reports in squamous cell carcinoma of the esophagus [44]. Here, PPZ differentially modulates adenosine receptor expression. In adenocarcinoma cells, A_2A_ and A_2B_ receptor expression was reduced, whereas squamous carcinoma cells displayed increased A_2A_ expression without changes in A_2B_ levels. Functional assays further demonstrated that antagonism of A_2A_ and A_2B_ receptors reversed PPZ‑induced growth inhibition exclusively in adenocarcinoma cells, confirming the involvement of these receptors in mediating PPZ’s antiproliferative effects in this histological subtype. These data are consistent with previous reports implicating A_2A_ and A_2B_ receptors in tumor growth, angiogenesis, and immune modulation [45].

Following PPZ treatment, modulation of the purinergic axis was observed, leading to alterations in extracellular adenosine availability. A previous study showed that increased adenosine concentrations may preferentially activate A_2A_ receptors, which are coupled to Gs proteins and stimulate cAMP production, leading to activation of the PKA/CREB pathway. In addition, A_2A_ receptor signaling can engage other pathways, including PI3K/AKT and MAPK/ERK, which are involved in cell proliferation, survival, and other cellular responses [46]. Therefore, the effects of PPZ may be related not only to changes in A_2A_ receptor expression but also to the activation of downstream signaling pathways associated with this receptor.

In addition, PPZ modulated CD73 expression under the tested conditions, suggesting that CD73 may be upregulated as a compensatory response. Notably, despite reducing tumor cell number and clonogenic capacity, PPZ did not alter AMPase activity. A previous study using the esophageal cancer cell line KYSE450 reported higher expression of E‑NTPDases 1 and 2 and ecto‑5′‑nucleotidase/CD73 compared with normal cells [10]. These findings highlight the complexity of CD73 regulation and underscore the need for further studies to elucidate the specific cell‑death or growth‑arrest pathways involved. While CD73 expression was reduced in adenocarcinoma cells and increased in squamous cells, enzymatic activity was not altered in either model. The increased CD73 expression observed in KYSE450 cells following pantoprazole treatment may represent an adaptive response aimed at preserving adenosinergic signaling. In this context, other components of the adenosine pathway may also contribute to this compensatory mechanism. CD39, which hydrolyzes extracellular ATP and ADP to AMP, thereby providing the substrate for CD73-mediated adenosine production, is a potential contributor. Previous studies have highlighted the coordinated regulation of CD39 and CD73 within the tumor microenvironment and have reported compensatory responses following modulation of adenosine pathway components [47]. However, because CD39 expression and activity were not evaluated in the present study, its role in the changes observed in KYSE450 cells remains speculative and warrants further investigation. This apparent discrepancy may reflect compensatory regulation in surviving cells or post‑translational modulation of enzyme function. Importantly, inhibition of CD73 with APCP abolished the effects observed with PPZ treatment.

Finally, PPZ reduced cell adhesion and MMP‑2 expression in adenocarcinoma cells but not in squamous carcinoma cells, further supporting a subtype‑specific impact on invasive and remodeling pathways. Given the role of the CD73–adenosine axis in regulating extracellular matrix interactions [48], these findings align with the broader modulation of adenosinergic signaling observed in OE33 cells. In fact, a study showed that CD73 expressed on fibroblasts interacts with EMMPRIN and is a required factor for MMP-2 production in co-cultures of sarcoma cells with fibroblasts [49]. Overall, PPZ suppressed proliferation and long‑term survival while modulating CD73‑dependent adenosine production, reinforcing the adenosinergic pathway as a relevant target in esophageal cancer. Collectively, our data indicate a shared molecular recognition mechanism for PPZ at both CD73 and P1 receptors, driven by conserved aromatic stacking and hydrogen‑bond interactions, supporting a multitarget modulation of the adenosinergic system. In conclusion, PPZ exerts antitumor effects in esophageal cancer through coordinated regulation of CD73 activity and A_2_ adenosine receptor signaling, with pronounced and selective effects in adenocarcinoma cells. These findings support the repositioning of PPIs, particularly PPZ, as potential adjuvant agents in esophageal adenocarcinoma and warrant further mechanistic and translational investigation.

A limitation of the present study is the relatively high concentrations of PPZ required to induce the observed effects in vitro. The concentrations tested (50–300 µM) are higher than the peak plasma concentrations expected following conventional clinical administration (~ 5–10 µM). However, comparable concentrations have been employed in previous in vitro studies investigating the direct anticancer effects of PPIs. For example, Feng et al. [28] reported that PPZ at 100 µg/mL (~ 231 µM) inhibited proliferation, self-renewal, and chemoresistance in gastric cancer stem cells, supporting the use of relatively high concentrations of PPZ in experimental models of cancer [50, 51]. Therefore, the findings in the present study should be interpreted as evidence of the biological activity and potential molecular mechanisms of PPZ in esophageal cancer cells, rather than as a direct indication of clinically achievable systemic effects.

Moreover, the clinical use of PPIs in oncology remains uncertain, as these agents may interact with various anticancer therapies, potentially altering their absorption, efficacy, and toxicity profiles [52]. Further studies using clinically relevant exposure conditions, pharmacokinetic assessments, and vivo models, followed ultimately by prospective clinical trials are warranted to determine whether the effects observed in vitro can be translated under physiological conditions and whether PPZ can be safely and effectively explored as an adjuvant therapeutic strategy.

## Methods

### Molecular docking

The prediction of protein–ligand complexes, as well as the estimation of IC₅₀ values, was performed using Boltz-2 v2.2.0 [30], a deep learning–based framework that predicts protein–ligand complex structures from sequence information and estimates small-molecule binding affinity. Target protein sequences were retrieved from the UniProt [53] database (CD73: P21589; adenosine receptor A_1_: P30542, A_2A_: P29274, A_2B_: P29275, A_3_: P0DMS8). The PPZ molecule was obtained from PubChem (CID 4679) in SMILES format. The parameters used in this study included the use of a default multiple sequence alignment (MSA) server for the automatic generation of evolutionary profiles [54], with a (i) maximum of 4096 MSA sequences, (ii) molecular dynamics as the prediction method, (iii) 25 recycling steps, (iv) 10 diffusion samples, (v) 400 affinity sampling steps, (vi) 50 affinity diffusion samples, and a (vii) maximum of 10 parallel samples. In addition, the *affinity_mw_correction* and *use_potentials* flags were enabled. The structure exhibiting the highest model prediction quality among the protein-ligand complexes was selected for further analysis. Intermolecular interactions between the protein structures and PPZ were identified using PoseView [55], allowing the characterization of hydrogen bonds, hydrophobic interactions, and π–π interactions between the ligand and residues of the predicted binding site. The PyMOL Molecular Graphics System (v3.0; Schrödinger, LLC) was used for figure manipulation and generation.

### Cell culture and treatments

To account for the biological heterogeneity of esophageal cancer, OE33 and KYSE450 cell lines were selected to represent the two major histological subtypes: esophageal adenocarcinoma (EAC) and squamous cell carcinoma (SCC), respectively. This comparative experimental design allowed us to investigate whether the antitumor effects of pantoprazole and its modulation of the adenosinergic system are shared across histological subtypes or exhibit subtype-specific responses. The cells were obtained through a donation from the National Cancer Institute (INCA). OE33 and KYSE450 cell lines were maintained in RPMI culture medium supplemented with 10% fetal bovine serum (FBS) and were maintained in an incubator at 37 °C with 95% relative humidity and 5% CO_2_. Based on previous studies reporting biological effects of PPIs in cancer cell models [50, 51], PPZ was initially tested at concentrations ranging from 50 to 300 µM. Following these preliminary experiments, 150 µM was selected as the concentration for the subsequent experiments. In the viability and proliferation assays, the cells were treated for 24 and 48 h with different concentrations of PPZ (50–300 µM) to characterize its effects or in combination with P1 receptor antagonists: ZM241385 (A_2A_) and MRS1754 (A_2B_) at a concentration of 1 µM. For the enzymatic activity assay, the cells were previously treated with PPZ (150 µM) for 24 h and then pre-incubated with APCP (1 µM). In the remaining experiments, cells were treated with PPZ (150 µM) for 24 h. All treatments used were purchased from Sigma-Aldrich (USA). Pantoprazole was diluted in water, and the P1 receptor antagonists were diluted in Dimethyl sulfoxide (DMSO).

### Cell viability - MTS assay

The effect of PPZ on cell viability was evaluated by the MTS assay. OE33 and KYSE450 cells were plated at a density of 3 × 10³ cells/well in a 96-well plate. The cells were treated with different concentrations of PPZ (50–300 µM) for 24–48 h. After this period, 20 µL of MTS (3-(4,5-dimethylthiazol-2-yl)-5-(3-carboximethoxyphenyl)-2-(4-sulfophenyl)-2 H-tetrazolium) was added to each well, and the cells were incubated for 3 h in a humidified incubator at 37 °C and 5% CO₂. Finally, the absorbance was quantified using a 96-well plate reader (Spectra Max M2e, Soft Max^®^ Pro 5, Molecular Devices) at 490 nm.

### Cell count

The effect of PPZ on cell proliferation was evaluated by cell counting using the Trypan Blue exclusion test. A total of 3 × 10^4^ (OE33 and KYSE450) cells per well were plated in 24-well plates and allowed to proliferate for 24 h. Cells were then treated with different concentrations of PPZ (50–300 µM) for 24–48 h. After treatment, the cells were trypsinized, mixed with Trypan Blue dye, and counted using a Neubauer chamber. Cells that become non-viable end up having a rupture in their membrane that causes them to incorporate the Trypan Blue dye. Thus, blue-stained cells were classified as dead, and unstained cells were considered viable. Additionally, to evaluate the effects of P1 receptor antagonists on cell proliferation, OE33 and KYSE450 cells were treated with PPZ (150 µM) and/or P1 antagonists (1 µM) for 24 h, followed by cell counting using the same procedure.

### Clonogenic assay

To evaluate the effects of PPZ treatment on OE33 and KYSE450 cell colony formation, the clonogenic assay was performed. Cells were plated at a density of 3 × 10^4^ cells per well in 24-well plates and, after 24 h, were treated with 150 µM of PPZ. 24 h post treatment, they were trypsinized and replated at a density of 700 (OE33) or 300 (KYSE450) cells per well in 12-well plates. Cells were then maintained in culture for 10 days, with medium replacement every 3 days. At the end of the experiment, the cells were washed with PBS, fixed with 4% paraformaldehyde for 20 min, and stained with gentian violet for 10 min. Subsequently, cells were washed twice with PBS and allowed to dry at room temperature. Images were acquired and, after 24 h, colonies were quantified using an inverted microscope (Olympus IX71, Olympus Corporation, Tokyo, Japan). Results are presented as the absolute number of colonies.

### Nuclear morphology analysis

The Nuclear Morphology Analysis (NMA) was performed to evaluate the effects of PPZ treatment on nuclear cell morphology, following the methodology described by [56]. Cells were plated at a density of 1.5 × 10^4^ cells per well in 24-well plates and, after 24 h, were treated with 150 µM of PPZ. After an additional 24 h, the cells were fixed with 4% paraformaldehyde, permeabilized with a solution of 0.3% Triton X-100 and stained with DAPI (300 nM). At least four images per well were acquired using an inverted microscope (Olympus IX71, Olympus Corporation, Tokyo, Japan). Nuclear features were quantified in the software Image Pro Plus 6.0 (IPP6), including area, aspect, area box, radius ratio, and roundness, and parameters were used to calculate the Nuclear Irregularity Index (NII) and to generate dispersion plots.

### Apoptosis analysis

Apoptosis was quantified using the annexin V-FITC Apoptosis Detection Kit (BD Biosciences) according to the manufacturer’s instructions. Briefly, 1.5 × 10^5^ cells/well were plated in 6-well plates and, after 24 h, treated with 150 µM PPZ. Following 24 h of treatment, the supernatant containing detached cells were collected together with the trypsinized adherent cells. After washing with cold PBS, cells were resuspended in a binding buffer and incubated with Annexin V and PI for 15 min at room temperature in the dark. Following incubation time, cells were analyzed in a BD FACSCANTO II flow cytometer with at least 10,000 events recorded per sample. Data were analysed using FlowJo Software (Tree Star). Cell populations were distinguished as follows: Annexin V⁻/PI⁻ (viable cells), Annexin V⁺/PI⁻ (early apoptotic cells), Annexin V⁺/PI⁺ (late apoptotic or necrotic cells), and Annexin V⁻/PI⁺ (necrotic cells).

### Cell adhesion assay

For the cell adhesion assay, 1.5 × 10^5^ cells/well were plated in 6-well plates, and after 24 h, treated with PPZ at a concentration of 150 µM. After 24 h of treatment, cells were trypsinized and counted in Neubauer chambers. 2 × 10^4^ cells/well were replated in 96-well plates and kept in the incubator for 3 h. After the incubation period, the medium was removed, and the cells were carefully washed with PBS to remove the unadhered cells. The adherent cells were fixed with 4% paraformaldehyde for 20 min, washed again with PBS, and then stained with 0.4% crystal violet for 10 min. The excess dye was removed by PBS washing. Subsequently, the dye bound to the cells was solubilized with 100 µL of 10% acetic acid, and the absorbance was quantified at 490 nm using a Victor X3 microplate reader (model 2030, PerkinElmer). The results were expressed as a percentage of adherent cells relative to the control group.

### Real time-PCR

Following 24 h of treatment with 150 µM of PPZ, the expression of P1 receptors (A_2A_ and A_2B_), CD73, and MMP-2 was analyzed using RT-qPCR. Total RNA was isolated using Trizol LS (Invitrogen), and cDNA was synthesized using the GoScript™ reverse transcription system kit (Promega), according to the manufacturer’s instructions. Quantitative PCR was performed with 50 ng of cDNA using SYBR Green I (Invitrogen) in a LineGene 9660 thermocycler (Bioer, China). Relative RNA expression levels were determined with the 2^−ΔΔCT^ method, and β2M was used as a reference gene. The primer sequences used for RT-qPCR are presented in Table 2.

**Table 2:** Primer sequences used for RT-qPCR analysis of P1 receptors, CD73, and MMP-2 in human esophageal cell lines

Gene	Forward primer		Reverse primer	
ADORA_2A_^a^	5′-CCT TCA TCT ACG CCT ACC G-3′	5′-TGG GAC TCT TGG GCA CTC-3′		
ADORA_2B_^a^	5′-CTC CAT CTT CAG CCT TCT GG-3′	5′-ACA AGG CAG CAG CTT TCA TT-3′		
MMP-2^b^	5′-CCC AGC GAC TCT AGA AAC ACA-3′	5′-GGG CCA CTA TTT CTC CGC TT-3′		
CD73^*^	5’-AGG GGT GTG GAC GTC GTG GT-3′	5’-CCC AGC AGG CAC CTC TTT GGA-3′		
β2M^b^	5’-CTA TCC AGC GTA CTC CAA AG-3′	5’-ACA AGT CTG AAT GCT CCA CT-3′		

^a^ [57], ^b^ [58], ^*^Designed by the authors

### Enzymatic activity assay

The enzymatic activity assay was performed to determine the AMP hydrolysis due to CD73 (ecto-5′- nucleotidase) activity. 3 × 10⁴ cells/well of OE33 and KYSE450 cells were plated in 24-well plates and treated for 24 h with 150 µM PPZ. Following treatment, the supernatant was removed, and the cells were washed with phosphate-free medium. Then, the cells were incubated for 30 min with the incubation medium containing 2 mM of AMP. The reaction was stopped by the addition of 150 µL of the supernatant to 150 µL of 10% trichloroacetic acid (TCA) kept on ice. We also evaluated the effect of APCP (adenosine 5’-(α, β-methylene) diphosphate), a CD73 inhibitor, on AMPase activity by pre-incubating the cells for 10 min with 1 µM APCP diluted in the incubation medium [59]. The release of inorganic phosphate (Pi) was determined by the Malachite Green method with spectrophotometric reading at 630 nm. Protein concentration was determined by the Coomassie Blue method, using bovine serum albumin (BSA) as a standard. After the enzymatic assay, 100 µL of 1 M NaOH was added to the wells containing the cells, which were frozen at − 20 °C. After 24 h, the cells were thawed and homogenized, and 50 µl of each sample was diluted in Coomassie Blue and measured in a spectrophotometer (595 nm). The results were expressed as nmol of Pi/min/mg of protein.

### Statistical analysis

The results are presented as the mean ± standard error of the mean (SEM). Statistical analyses were performed using Student’s t-test, one-way analysis of variance (one-way ANOVA), or two-way analysis of variance (two-way ANOVA). P-values less than 0.05 were considered indicative of significance. The graphs were created using GraphPad Prism^®^ 7.0 software. All experiments were independently performed at least three times (*n* ≥ 3), in triplicate.

## Supplementary Information

Below is the link to the electronic supplementary material.

Supplementary Material 1

## Data Availability

Data will be made available on request.
