# Supplementary material for: Pantoprazole as a potential repurposed drug targeting the adenosinergic system in esophageal cancer
Source: Med Chem Res. 2026 Sep 25;35(10):2094–108. doi: 10.1007/s00044-026-03618-2 (PMC13668930; doi:10.1007/s00044-026-03618-2)
Supplement: Supplementary file 1 [file 44_2026_3618_MOESM1_ESM.docx]

**Sup. Fig. S1 Structural superposition of the predicted models with experimental structures of adenosine targets and docking of pantoprazole (PPZ) into the orthosteric binding site.** **(a)** CD73, **(b)** A_1_ , **(c)** A_2A_ , **(d)** A_2B_, and **(e)** A_3_. Predicted structural models are represented in pink, while crystallographic structures deposited in PDB are represented in grey. The highlighted panels show the binding site of each target with predicted PPZ (pink) docked and superimposed onto the original co-crystallized ligands (gray). PDB codes used for validation: CD73 (6TVE, 9HJ4, 7PBY, 7PB5, 7PCP), A_1_ (5UEN, 5N2S, 6D9H, 7LD3, 7LD4), A_2A_ (5NM4, 5IU4, 9H37, 9H2X, 4EIY), A_2B_ (7XY6, 7XY7, 8HDO, 8HDP), and A_3_  (8X16, 8X17, 9EBH, 9EBI, 9EHS).

**Sup. Fig. S2 Representative images of nuclear morphology assessed by DAPI staining. (a, c)** OE33 and KYSE450 control groups and **(b, d)** OE33 and KYSE450 after 24 h treatment with 150 μM PPZ. On **(b),** it is possible to see the presence of condensed nuclear structures within the cell nucleus, as indicated by the red arrows.

**Sup. Fig. S3 Apoptosis assessment by Annexin V/PI staining after PPZ treatment. (a)** OE33 and **(b)** KYSE450 were treated with 150 μM PPZ and after 24 h were analysed by flow cytometry. Bars represent the percentage of cells in early apoptosis (annexin V⁺/PI⁻, black) and late apoptosis/necrosis (annexin V⁺/PI⁺, grey). Data were analysed by Student’s *t*-test expressed as mean ± SEM of 3 independent experiments. *p < 0.05, **p < 0.01 versus the corresponding control group.
